# Transcriptome Expression Profiling in Response to Drought Stress in *Paulownia australis*

**DOI:** 10.3390/ijms15034583

**Published:** 2014-03-17

**Authors:** Yanpeng Dong, Guoqiang Fan, Zhenli Zhao, Minjie Deng

**Affiliations:** 1 Institute of Paulownia, Henan Agricultural University, 95 Wenhua Road, Jinshui Area, Zhengzhou 450002, Henan, China; E-Mails: dongdyp@163.com (Y.D.); zhaozl@126.com (Z.Z.); 2 College of Forestry, Henan Agricultural University, 95 Wenhua Road, Jinshui Area, Zhengzhou 450002, Henan, China; E-Mail: dengmj1980@126.com

**Keywords:** drought, diploid, autotetraploid, transcriptome

## Abstract

The response and adaptation to drought remains poorly understood for *Paulownia australis*. To investigate this issue, transcriptome profiling of four *P. australis* accessions (two diploid and the other two autotetraploid) under water stress condition were studied using Illumina Genome Analyzer II*x* analysis. The current study aimed to identify genes of *P. australis* metabolism pathways that might be involved in this plant’s response to water deficit. Potted seedlings were subjected to well-watered conditions and drought stress, respectively. More than 290 million raw transcript reads were assembled into 111,660 unigenes, with a mean length of 1013 bp. Clusters of orthologous groups, gene ontology and the Kyoto Encyclopedia of Genes and Genomes annotations analyses were performed on the unigenes. Many differentially expressed genes and several metabolic pathways were identified. Quantitative real-time polymerase chain reaction was used to verify the expression patterns of 14 genes. Our study identified altered gene expression in *P. australis* induced by drought stress and provided a comprehensive map of drought-responsive genes and pathways in this species. To our knowledge, this is the first publicly available global transcriptome study of *P. australis*. This study provides a valuable genetic resource for this species.

## 1. Introduction

Adverse environmental influences affect the survival, reproductive biology, stability and productivity of plants [1]. Plants are frequently subjected to various stresses, of which drought is the most common and devastating one. Water scarcity adversely affects plants on several levels. First, it causes reduced cell expansion and growth, and with progressive water deficit, photosynthesis is affected. At the microscopic level, a reduction in the hydration of the membranes and proteins can cause damage to these components accompanied by increased reactive oxygen species (ROS) [2].

Fortunately, plants have evolved sophisticated mechanisms to cope with such stress, ranging from physiological and biochemical responses to molecular and genetic changes [3]. As soon as the leaf-to-air vapor pressure changes, plant leaves close their stomata instantly to reduce water loss through transpiration [4]. This response is induced by phytohormone abscisic acid (ABA). The key role of ABA in regulating gene expression in response to drought is well described [5]. With progressive water loss, the leaves start to wilt to protect the photosynthetic machinery [6]. Cyclical electron flow around photosystem I (PSI) [7], as well as an increase in thermal dissipation in the photosystem II (PSII) antennae [8] are involved in this process. The water content of cells is then maintained by synthesizing and accumulating various groups of small molecule compounds, including soluble carbohydrates, polyols, amino acids or amides, and quaternary ammonium compounds [9]. Thus, the stress response and adaptation of a plant undergoing drought conditions is a complex network, and a cascade of genes is involved [10]. Identifying the genes related to adaptation to drought stress is of prime importance.

Before the emergence of current technologies for expression analysis and next-generation sequencing techniques, only a few drought-related genes had been identified. In a pioneering work in 1996, four drought-induced cDNAs were identified in *Pinus taeda* [11]. After that, 48 genes that might be related to the response to water stress in *P. pinaster* were identified using a cDNA-amplified fragment length polymorphism and reverse northern blotting [12]. Presently, more accurate technologies, such as suppression subtractive hybridization (SSH) [13] and microarrays [14], which are capable of identifying many differentially expressed genes at a time, have provided efficient ways to explore the molecular mechanisms of a plant’s response and adaptation to adverse environments. In addition, high-throughput sequencing techniques have become more affordable; thus, analysis of drought-induced genome-wide transcription by RNA-seq, for example, is currently feasible. This method has been used to efficiently mine responsive genes under drought stress in model plants and agronomically important crop species [5].

The plant response to drought is a polygenic trait that involves the activation of a cascade of genes, whose functions range from water deficiency perception to stress signal transmission. These genes can be categorized into two major groups based on their putative biological function in the process of drought response. The first one contains genes encoding regulatory proteins that regulate the transduction of the stress signal and modulate the expressions of other genes. These proteins include protein kinases and phosphatases, transcription factors (such as NAC, bHLH, WRKY, AP2/EREBP, DREB/CBF, MYB, zinc finger and bZIP/AREB/ABF proteins) and phytohormones (such as ABA, salicylic acid, jasmonic acid and ethylene) [5,15]. The other group comprises genes encoding proteins that act as protectors of cells against water scarcity. Their functions include passive transport across members, water transport systems, energy requiring systems, accumulation of compatible solutes (e.g., sugar and proline) and protection of cell components against dehydration and damage by ROS.

*Paulownia australis* is a fast-growing, deciduous hardwood species native to China, whose history encompasses 2,000 years and has been introduced to other countries [5,16,17]. Its rapid growth, high ignition point, rot resistance, straight grain, knot-free wood with a satiny luster and high biomass production have meant that Paulownia has been widely used in forestation, the biofuel industry, as well as in paper or furniture making, as plywood, and for toys and musical instruments [18]. Paulownia is mainly planted in areas where irrigation water is limited or the soil is salinized [19]. Such environmental conditions have endowed this species with excellent tolerance to drought and extreme soils, which is important in land reclamation of surface-mined land [20].

To understand the molecular mechanisms of *Paulownia*’s response to drought, the transcriptomes of *P. australis* were assembled. Since biological replication of high throughput sequencing technology is becoming a concern for researchers, many researchers are now taking this into account when designing their experiments. For example, Mou *et al*. [21] performed a transcriptomic analysis of both samples from tissue cultured plants (tissue cultured healthy plants (TH) and tissue cultured disease plants (TD)) and field grown plants (field grown diseased plants (FD) and field grown healthy plants (FH)). In this study, diploid and autotetraploid *P. australis* under well-watered conditions and drought treated conditions were analyzed. The autotetraploid was another ideal material for the research of the drought response of Paulownia besides the natural species diploid *P. australis*. The two different types of *P. australis* were derived from the same tissue culture and could be justified as biological replicates. For these, 265,017,594 clean reads were assembled into 111,660 unigenes with a mean length of 1013 bp. Clusters of orthologous groups (COG), Gene Ontology (GO) and the Kyoto Encyclopedia of Genes and Genomes (KEGG) annotations were performed for these unigenes. Differentially expressed genes (*DEG*s) involved in the response to drought were identified and analyzed in detail. Quantitative Real-Time PCR (qRT-PCR) was performed to verify the expression patterns of 14 *DEG*s. To the best our knowledge, this is the first report of the transcriptome of *P. australis*. The data provide a resource of gene sequences for this species. The genes and pathways related to the drought response represent putative candidates for manipulation to improve the adaptation to drought of this plant or even other species.

## 2. Results and Discussion

### 2.1. Physiological Responses of Diploid and Tetraploid Paulownia to Drought Stress

**Figure 1.**
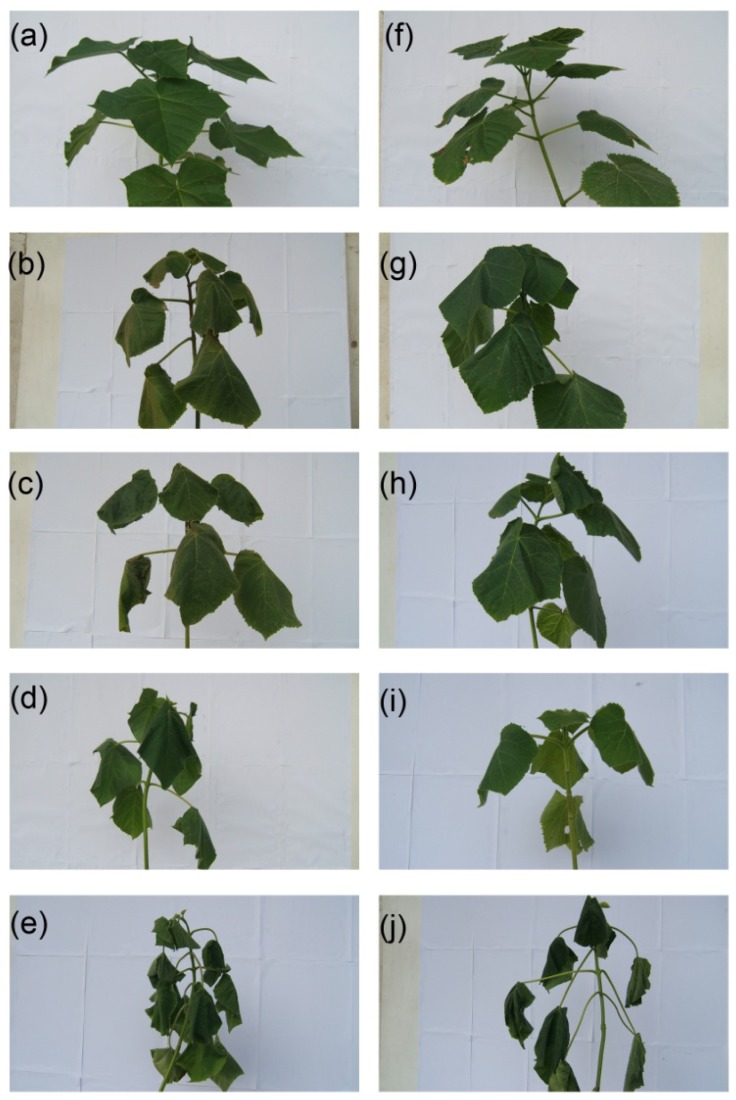
Physiological features of diploids and tetraploids in response to drought. PA2 represents diploid *P. australis*, PA4 represents autotetraploid *P. australis*. (**a**) PA2, well-watered diploid, 75% relative soil water content; (**b**) PA2T-1, three-day drought treated diploid, 25% relative soil water content; (**c**) PA2T-2, six-day drought treated diploid, 25% relative soil water content; (**d**) PA2T-3, nine-day drought treated diploid, 25% relative soil water content; (**e**) PA2T, 12-day drought treated diploid, 25% relative soil water content; (**f**) PA4, well-watered tetraploid, 75% relative soil water content; (**g**) PA4T-1, three-day drought treated tetraploid, 25% relative soil water content; (**h**) PA4T-2, six-day drought treated tetraploid, 25% relative soil water content; (**i**) PA4T-3, nine-day drought treated tetraploid, 25% relative soil water content; (**j**) PA4T, 12-day drought treated tetraploid, 25% relative soil water content.

With 25% and 75% relative soil water contents, the physiological responses of tetraploid and diploid *Paulownia* plants to drought stress tolerance were studied. In the tetraploid and diploid *Paulownia* plants, the changing trends of leaf physiological and biochemical indexes were consistent with the aggravation of drought stress (Figure 1).

**Figure 2.**
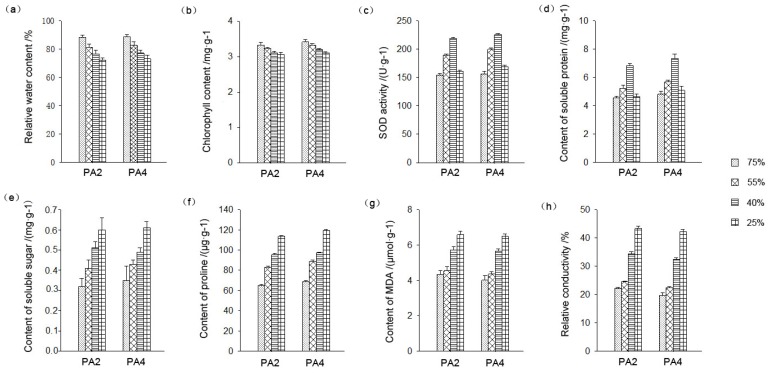
Effects of drought stress on *P. australis* physiology. PA2 represents diploid *P. australis*, PA4 represents autotetraploid *P. australis*, 75%, 55%, 40% and 25% relative soil water contents were used. (**a**) Effect of drought stress on leaf relative water content; (**b**) Effect of drought stress on leaf chlorophyll content; (**c**) Effect of drought stress on leaf superoxide dismutase (SOD) activities; (**d**) Effect of drought stress on leaf soluble protein content; (**e**) Effect of drought stress on leaf soluble sugar content; (**f**) Effect of drought stress on leaf proline content; (**g**) Effect of drought stress on leaf malondialdehyde (MDA) content; (**h**) Effect of drought stress on leaf relative conductivity content.

The water and chlorophyll contents of the leaves decreased during drought stress. The water and chlorophyll contents of the drought-treated plant leaves were lower than those of the well-watered plants in both the tetraploids and diploids. The relative conductivity and malondialdehyde (MDA) content increased during drought stress. The superoxide dismutase (SOD) activity and soluble protein content initially increased and then decreased. The soluble sugar and proline contents increased during drought stress, with higher levels in the tetraploids than in the diploids (Figure 2) [22].

### 2.2. Illumina Paired-End Sequencing and the *De Novo* Assembly of Paulownia

To generate a broad database of genes involved in the drought response and tolerance of *P. australis*, the RNA from the leaves of seedlings of a drought-treated and well-watered diploid (PA2T and PA2) and autotetraploid (PA4T and PA4) *P. australis* was extracted. Using Illumina GAII*x* sequencing technology, over 290 million raw reads with a mean length of 110 bp were obtained from the four libraries. After filtering out the adaptor sequences, ambiguous reads and low-quality sequences, 265 million clean reads representing 23.8 Gb with a Q20 (quality error rate of 0.01) percentage of 97.3% and an *N* (nucleoside) percentage of 0 remained. These clean data were then assembled *de novo* by the Trinity program, which generated 602,121 contigs with an average length of 312 bp and an N50 of 488 bp. Trinity was then used to assemble these contigs into a library of 111,660 unigenes. The unigenes could be divided into two classes: clusters and singletons. Clusters comprise several unigenes, whose similarity to one another is more than 70%. Among these, 56,446 clusters and 55,214 singletons were assembled. The clusters and singletons had a mean length of 1013 bp with an N50 of 1667 bp: the total length of these unigenes was 113,092,718 nt (Table 1). Among them, 72,866 unigenes (65.26%) were longer than 500 bp, and 44,763 unigenes (40.09%) were longer than 1000 bp. The length distribution of the unigenes is shown in Figure S1. The gap distributions were also analyzed for both contigs and unigenes: none of them had gaps.

### 2.3. Annotation of the Paulownia Unigenes against the Public Databases

**Figure 3.**
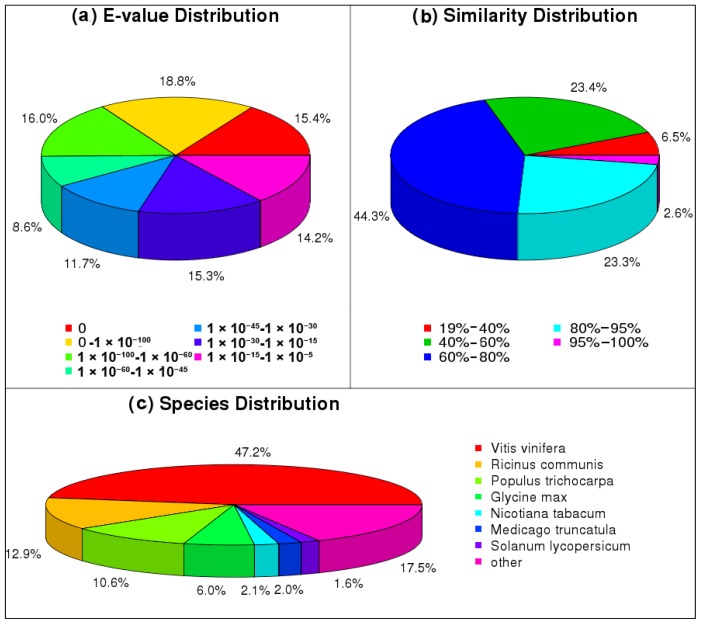
*E*-value distribution (**a**), similarity distribution (**b**) and species distribution (**c**) of the BLAST matches of the transcriptome unigenes. This figure shows the distributions of unigene BLASTX matches against the Nr protein database (cutoff value: *E* < 1.0 × 10^−5^) and the proportions for each species.

For annotation, BLASTX was used to align the unigenes against sequences in six public databases: non-redundant protein database (Nr) (ftp://ftp.ncbi.nih.gov/blast/db/nr), nucleotide sequence database (Nt) (ftp://ftp.ncbi.nih.gov/blast/db/nt), Swiss-Prot (http://www.ebi.ac.uk/swissprot/), KEGG (http://www.genome.jp/kegg/) and COG (http://www.ncbi.nlm.nih.gov/COG/), using an *E*-value threshold of 1.0 × 10^−5^. Among the 111,660 unigenes, the highest number, 76,322 (68.35%), had homologous sequences in the Nr database (Table S1), followed by Nt (67,551, 60.50%), GO (63,517, 56.88%), Swiss-Prot (49,904, 44.69%), KEGG (46,740, 41.86%) and COG (31,550, 28.26%). Altogether, 79,031 (70.78%) unigenes were identified against the six databases. In the alignment against the Nr database, the E-value distribution of the top hits revealed that 34.2% of the matched sequences had a significant similarity with an *E*-value of less than 1.0 × 10^−100^. The majority of the *E*-values were distributed in the range of 1.0 × 10^−15^ to 1.0 × 10^−100^ (Figure 3A). The similarity distribution showed that 70.2% of the hits had a similarity over 60%, whereas 91.0% of the unigenes had a similarity ranging from 40% to 95% (Figure 3B). The species distribution revealed that 47.2% of the hits matched sequences from *Vitis vinifera*, followed by *Ricinus communis* (12.9%), *Populus trichocarpa* (10.6%), *Glycine max* (6.0%), *Nicotiana tabacum* (2.1%), *Medicago truncatula* (2.0%), *Solanum lycopersicum* (1.6%) and other species (17.5%) (Figure 3C).

### 2.4. Functional Analysis of the Unigenes of Paulownia Leaves

**Figure 4.**
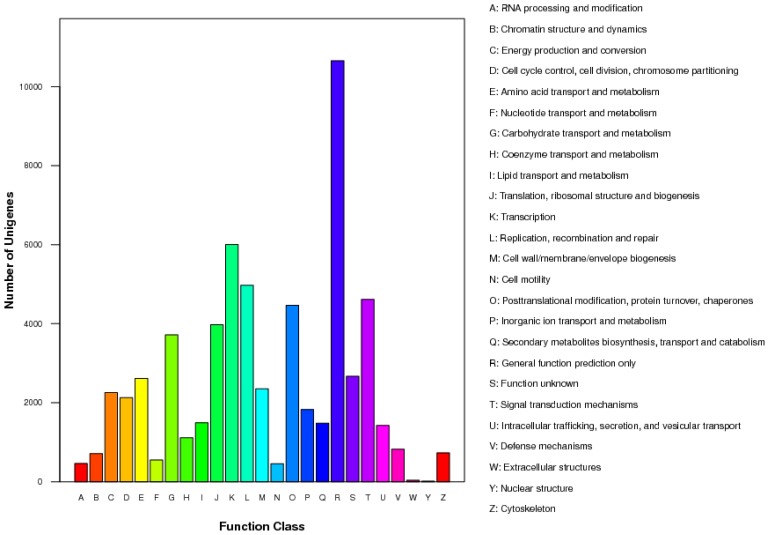
Classification of the clusters of orthologous groups (COG) for the transcriptome of *P. australis*. 31,550 unigenes (8.26% of the total) were annotated and divided into 25 specific categories.

After searching against the COG database, 31,550 unigenes (28.26%) were categorized into 25 specific functional groups (Figure 4). The cluster of “General function prediction only” was the largest category (10,653, 9.54%), followed by “Transcription” (6002, 5.38%), “Replication, recombination and repair” (4972, 4.45%) and “Signal transduction mechanisms” (4613, 4.13%). By contrast, the clusters for “Nuclear structure” (15, 0.013%), “Extracellular structures” (38, 0.034%) and “Cell motility” (457, 0.41%) were the smallest categories. The detailed information is shown in Figure 4.

**Figure 5.**
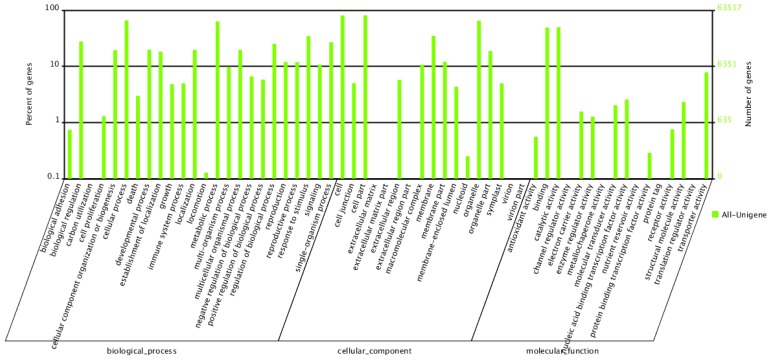
Classification of the gene ontology (GO) for the transcriptome of *P. australis*; 63,517 unigenes (56.88% of total) were categorized into 58 function groups.

To identify domain-based annotations, unigenes were assigned to the GO database with an *E*-value of 1.0 × 10^−5^. The result showed that 63,517 (56.88%) of the unigenes had an entry description in GO according to the Blast2GO program and were categorized into 58 functional categories. Most categories had more than one unigene. Among them, “Cell” and “Cell part” were the two largest groups, comprising 51,383 unigenes (46.01%), followed by “Cellular process” (41,902, 37.5%) and “organelle” (41,324, 37.0%). Based on the three basic functional categories of GO, the largest group in the “Molecular function” was “catalytic activity” (31,536, 28.24%). The detailed information, generated using Web Gene Ontology Annotation Plot (WEGO), is shown in Figure 5.

### 2.5. Metabolic Pathway Analysis of Paulownia Leaf Transcripts

The potential involvement of the *P. australis* unigenes in metabolic pathways was investigated using the KEGG annotation system. Among 111,660 assembled unigenes, 46,740 homologous unigenes (41.86%) were mapped to 128 KEGG reference pathways. Among the pathways, the most significantly abundant term was “Metabolic pathways” (10,918, ko01100), followed by “Biosynthesis of secondary metabolites” (5312, ko01110), “Plant-pathogen interaction” (2819, 6.03%), “Plant hormone signal transduction” (2347, ko04075), “Spliceosome” (1811, ko03040) and “RNA transport” (1671, ko03013). The term representing the least number of unigenes was betalain synthesis (ko00965), which contained only three unigenes (Table S2). After alignment with these databases, 78,502 coding sequences (CDSs) were predicted, of which 76,427 were inferred by BLASTX and 2075 by ESTScan [23]. The length distribution of the CDSs in Blastx is shown in Figure S2, and the CDSs generated by ESTScan are shown in Figure S3.

### 2.6. DEGs Related to Drought Response

**Figure 6.**
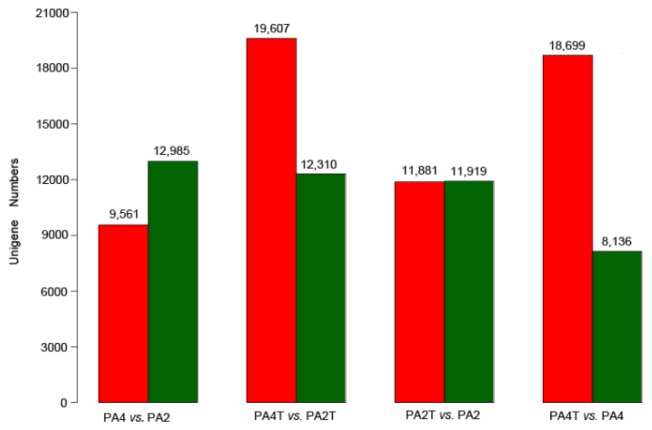
The statistics of differentially expressed genes in each pairwise comparison. Red bars represent the up-regulated genes, while green bars represent the down-regulated ones. PA4T, 12-day drought treated tetraploid; PA4, well-watered tetraploid. PA2T, 12-day drought treated diploid. PA2, well-watered diploid. A threshold of *FDR* < 0.001 and an absolute value of log2 ratio > 1 were used to judge the significance of the differences in gene expression.

To identify essential genes or pathways and further understand the biology of the drought response and tolerance, we compared the drought-treated accession with the well-watered accession of *P. australis*, both in the diploid and its autotetraploid. Genes with a threshold of false discovery rate, *FDR* < 0.001, and an absolute value of log2 ratio > 1 were considered differentially expressed ones between the two accessions. Such genes between the two accessions were identified in each comparison, and the comparison of the expression levels for the diploid and autotetraploid is shown in Figure 6. Among the 111,660 unigenes, 23,800 (21.31%) were differentially expressed between the diploid accessions and 22,576 (20.22%) for the autotetraploid accessions. Among these differentially expressed transcripts, 9561 were up-regulated and 12,985 were down-regulated in autotetraploid *P. australis*. The top 100 up-regulated and down-regulated genes are shown in Tables S3 and S4. Genes differentially expressed in the PA4 *vs.* PA2 comparison were also retrieved (Table S5). To retrieve consistent drought response genes, genes up-regulated or down-regulated with a threshold of *FDR* < 0.001 and an absolute value of log2 ratio > 2 in both comparisons were selected. One thousand three hundred and eighty genes were picked out, in which, 445 were up-regulated and 935 were down-regulated (Table S6). To evaluate the potential function of differentially expressed transcripts, their GO categories were determined. The detailed information is shown in Figures S4 and S5. GO categories of the consistent *DEG*s in both comparisons were also evaluated (Figure S6). Statistical analysis of biological process, cell component and molecular function of the *DEG*s in both the PA2T *vs.* PA2 and PA4T *vs.* PA4 comparisons are shown in Table 2.

Furthermore, we mapped all the differentially expressed transcripts to the terms in the KEGG database and compared this with the whole transcriptome background to identify genes involved in significantly enriched metabolic pathways. For the diploid, 23,800 differentially expressed unigenes were assigned to 128 KEGG pathways on the third level. Unigenes of these pathways were significantly enriched with “Metabolic pathways”, “Photosynthesis”, “Biosynthesis of secondary metabolites”, “Starch and sucrose metabolism” and “Phenylpropanoid biosynthesis” as the top four pathways. In the autotetraploid, 22,576 differentially expressed unigenes were assigned to 127 KEGG pathways. The top five pathways were “Metabolic pathways”, “Biosynthesis of secondary metabolites”, “Ribosome”, “Glyoxylate and dicarboxylate metabolism” and “Plant-pathogen interaction”. The detailed information is shown in Tables S7 and S8. In the consistently up- or down-expressed genes retrieved from both comparisons (with a threshold of *FDR* < 0.001 and an absolute value of log2 ratio > 2), 673 differentially expressed genes were assigned to 103 KEGG pathways (Table 3).

### 2.7. qRT-PCR Verification of Genes Related to Drought Response in Paulownia Leaves

**Figure 7.**
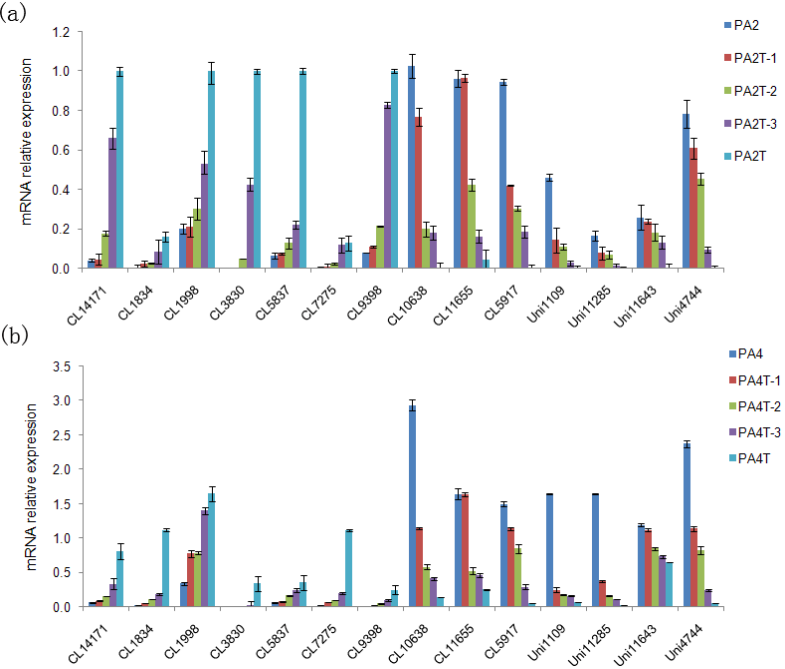
Quantitative Real-Time PCR (qRT-PCR) analysis of 14 potential drought response *DEG*s. CL14171, laccase-14. CL1834, asparagine synthetase. CL1998, alanine-glyoxylate aminotransferase 2. CL3830, dehydrin. CL5837, inositol oxygenase 1. CL7275, flavonoid glycosyltransferase. CL9398, short chain alcohol dehydrogenase. CL10638, ribulose bisphosphate carboxylase. CL11655, mitogen-activated protein kinase kinase. CL5917, carbonic anhydrase. Unigene1109, cytochrome P450 76A2. Unigene11285, geraniol 10-hydroxylase. Unigene11643, disease resistance response protein 206. Unigene4744, photosystem I reaction center subunit X psaK. 18S rRNA was used as the internal reference gene, and other samples were normalized accordingly. The standard error of the mean for three technical replicates is represented by the error bars. (**a**) PA2T, 12-day drought treated diploid; PA2T-3, nine-day drought treated diploid, PA2T-2; six-day drought treated diploid; PA2T-1, three-day drought treated diploid; PA2, well-watered diploid; (**b**) PA4T, 12-day drought treated tetraploid; PA4T-3, nine-day drought treated tetraploid; PA4T-2, six-day drought treated tetraploid; PA4T-1, three-day drought treated tetraploid; PA4, well-watered tetraploid.

The expression profiles of 14 differentially expressed unigenes from the leaves of the drought-treated accessions and the well-watered accessions were assessed by qRT-PCR to confirm the results of the transcriptome analysis. The results showed that the up- or down-expression profiles of these 14 genes agreed with the RNA-seq result. In both the tetraploid and diploid *Paulownia* plants, the changing trends of gene expression were also consistent with the aggravation of drought stress (Figure 7). The results indicated the validity of the sequencing method.

### 2.8. Discussion

In the present study, a *de novo* assembly by Trinity was performed to generate a reliable and substantial genome-wide transcript dataset of the leaves of diploid and autotetraploid *P. australis* sequenced by the Illumina Solexa platform GAII*x*. More than 65% of the unigenes were longer than 500 bp, and the gap rate was 0. As in most previous studies, the mean length of the contigs (312 bp) was much shorter than that of the unigenes (1013 bp). Of the 111,660 unigenes, 79,031 (70.78%) could be aligned to sequences in six public databases. The unmatched unigenes are most likely to represent *Paulownia*-specific genes. There were 78,502 CDSs predicted, among which there was over 603-fold coverage of the 130 *Paulownia* protein sequences available in the National Center for Biotechnology Information (NCBI) GenBank database. The results suggested that this sequencing and assembly was of a high quality. In the KEGG analysis, “metabolic pathways” (10,918 unigenes) was the most highly represented category in the transcriptome of *P. australis* (23.36%), followed by “biosynthesis of secondary metabolites”.

After comparison of the drought-treated accessions and the well-watered accessions of *P. australis*, we narrowed the scope of the *DEG* group to identify genes that might be involved in drought response and adaptation. The two different types of *P. australis* (diploid and autotetraploid) were derived from the same tissue culture, which could be justifying as biological replicates in this study. Common genes that up- or down-expressed consistently in both diploid and tetraploid plants under drought condition have been retrieved (Table S6). Bioinformatics analyses were done to these genes.

There were 488 DEGs in the starch and sucrose metabolism pathway in the diploid comparison compared with 358 in the autotetraploid comparison. The genes probably involved in glucose and starch syntheses were found to be down-regulated in the drought-treated accessions. Meanwhile, a gene encoding a putative soluble invertase (CL6075.Contig1_All, gi|294612070|gb|ADF27779.1|) was down-regulated in *P. australis* in response to drought. Five putative glucose transmembrane transporters were up-regulated in both the diploid and the autotetraploid. The generating and scavenging pathways of ROS and many putative related genes, including catalases, peroxisomes, superoxide dismutases (SODs) and glutathione, were identified in the comparisons of the drought-treated and well-watered (Tables S6–S8) samples.

It seemed that crucial stress signaling events were induced in the drought-stressed *Paulownia* leaf meristem. The drought-related phytohormone ABA appears to increase in the drought-treated leaf [24,25]. The rates of ABA biosynthesis and catabolism are critical in determining the accumulation of ABA, as well as the strength of the response. Although a variety of organisms synthesize ABA, the carotenoid pathway in angiosperms is the only defined pathway for ABA biosynthesis [26]. In our study, 11 genes (e.g., *PP2C*, *SnRK2*) were up-regulated in the carotenoid biosynthesis pathway (Table 3), which may be the response for accumulating extra ABA. Then, the extra ABA may have acted to repress the expression of the soluble invertase in both the drought-treated diploid and the autotetraploid. With respect to its metabolic function, the repression of the putative soluble invertase gene might lower the level of the protein in the cell wall. It was reported that in drought-stressed maize ovary tissue, invertase mRNA and protein levels were down-regulated [27]. In addition, less substrate was available for the mitochondrial hexokinase to generate ADP to maintain coupled electron transport [28]. At the same time, the available glucose decreases because of the differential expression of glucose transmembrane transporters, which could be sensed by a signaling molecule, as is the case for hexokinase. As a result, the metabolic function of this invertase enzyme is abnormal. Previous studies have illustrated the positive influence of low-sugar signaling. Under low sugar signaling, the ROS level in the mitochondria increased to a level high enough to repress the expression of genes encoding ROS-scavenging related genes, both in the mitochondrion and the cytosol. Increased levels of catalases and peroxisomes were not sufficient to detoxify ROS that accumulated in the drought-treated leaves [29,30]. The same phenomenon, including ABA action and disruption of hexose-sucrose ratios induced by the repression of invertases, was also found in the leaves of other species, and it is reported that it can bring about a biological process similar to the ABA-mediated acceleration of senescence [31]. Several genes involved in ABA catabolism have been identified in *Arabidopsis*. To date, only *CYP707A* genes for 8′-hydroxylation of ABA have been reported in *Arabidopsis* [32], and these genes are involved in drought tolerance [33], seed dormancy, as well as germination [34]. It is the main irreversible catabolic pathway in angiosperms [26]. In our study, three *CYP707A*-like genes were found to be down-regulated in both PA2T and PA4T (Table S6). Another catabolic pathway is the conjugation of ABA to glucose-ester (GE), which is subsequently sequestered into vacuoles [35,36]. ABA-GE is reversibly converted to ABA by beta-glucosidase (BG) in *Arabidopsis*. To date, two *Arabidopsis* BG genes, endoplasmic reticulum localized AtBG1 and vacuole localized AtBG2, have been reported to catalyze ABA-GE hydrolysis [37,38]. In the consistently expressed genes in both comparisons of our study, one gene (Unigene16296_All, gi|16903208|gb|AAL27856.1|) encoding complete CDs of a putative beta-glucosidase (bglc) mRNA was found to be down-regulated.

Some of the DEGs were annotated as being involved in crosstalk among ABA and other important phytohormones. It has been reported that ethylene is involved in almost all kinds of abiotic stress, including drought [39]. Genes that putatively encode key enzymes in the ethylene biosynthesis pathway, as well as auxin and brassinosteroid biosynthesis pathways, were found to be elevated in both PA2T and PA4T (Tables 3 and S6). A series of genes encoding enzymes in the zeatin biosynthesis pathway were also found to be elevated in both PA2T and PA4T accessions (Tables 3 and S6). Although so many genes were found to be involved in ABA related drought response, how drought stress is perceived by ABA is still an issue that needs to be investigated further.

To verify our hypothesis, qRT-PCR was used to analyze the expression profiles of 14 unigenes related to these processes. The results showed that 14 of the sequences had the same up- or down-expression pattern found in the transcriptome analysis. These genes, together with other related genes, represent candidate drought-responsive genes. In addition to the annotated sequences, there were many un-annotated unigenes that might represent *Paulownia*-specific genes. These should be explored in future research.

## 3. Experimental Section

### 3.1. Materials

All the biological materials were obtained from the Institute of *Paulownia*, Henan Agricultural University, Zhengzhou, Henan Province, China. The tissue culture seedlings of diploid and tetraploid *P. australis* were cultured for 30 days before being clipped from the roots. Samples were transferred into nutrition blocks containing ordinary garden soil for 30 days. Samples with the same growing consistency were then transferred into nutrition pots of 30-cm diameters with trays underneath. Each pot was filled with the same amount of ordinary garden soil, one for each plant. After 50 days, tissue culture seedlings with the same growing consistency were subjected to drought conditions in a water-controlled experiment according to the method of Zhang *et al.* [22]. Diploid and tetraploid *Paulownia* with 25% and 75% relative soil water contents were named PA2T and PA2, PA4T and PA4, respectively. Three individuals for PA2T or PA2 and twelve individuals for PA4T or PA4 were prepared.

### 3.2. Physiological Responses of Diploid and Tetraploid Paulownia to Drought Stress

After 3, 6, 9 and 12 days (wilting state), respectively, the second pairs of leaves from the growing apex of the young sprout of the plants were picked from the drought-treated samples. The corresponding diploid leaf samples were renamed PA2T-1, PA2T-2, PA2T-3 and PA2T, respectively, while the corresponding tetraploid ones were renamed PA4T-1, PA4T-2, PA4T-3 and PA4T, respectively. The well-watered samples were picked after 12 days only. At least three parallel samples were prepared for each condition. The water and chlorophyll contents were measured according to the methods of Bao [40] and An *et al.* [41], respectively. The relative conductivity, the MDA content, the SOD activity, the soluble protein content and the proline content were measured according to the methods of Li [42]. The physiological responses of tetraploids and diploid *Paulownia* plants to drought stress tolerance were reported by Zhang *et al.* [22].

### 3.3. Construction of cDNA Libraries of Paulownia

After 12 days of 75% (well-watered) or 25% relative soil water contents treatment, for each condition, approximately 8 mg of leaves from three samples were homogenized in liquid nitrogen with a pestle. Total RNA was extracted from the cells using TRIzol reagent (Invitrogen, Carlsbad, CA, USA), followed by RNA purification using an RNeasy Mini Elute Cleanup Kit (Qiagen, Dusseldorf, Germany), according to the manufacturer’s protocol. A NanoVue UV-Vis spectrophotometer (GE Healthcare Bio-Science, Uppsala, Sweden) was used to quantify the RNA by measuring the absorbance at 260, 230 and 280 nm. Absorbance ratios of optical density (OD)_260/280_ and OD_260/230_ were taken into account for assessing the purity of all RNA samples. The integrity of the RNA was checked by 1% agarose gel electrophoresis. After total RNA extraction and DNase I treatment, magnetic beads with oligo (dT) probes were used to isolate mRNA. The mRNA was mixed with the fragmentation buffer (9.8% potassium acetate, 7.2% Tris–acetate, 6.4% magnesium acetate) and fragmented into short fragments. cDNA was then synthesized using the mRNA fragments as templates. The short cDNA fragments were purified and dissolved in ethidium bromide (EB) buffer (Tris–Cl 10 mM, pH 8.5 with 0.1% Tween 20) for end reparation and single nucleotide A (adenine) addition. The short fragments were then connected to adapter sequences. Suitable fragments were selected for PCR amplification as templates. During the quality control (QC) steps, an Agilent 2100 Bioanalyzer (Agilent Technologies, Palo Alto, CA, USA) and an ABI Step One Plus Real-Time PCR System (ABI, New York, NY, USA) were used to quantify and quality check the sample library. Finally, the library could be sequenced using an IlluminaHiSeq™ 2000 (Illumina, San Diego, CA, USA) or other sequencer when necessary.

### 3.4. Bioinformatic Analysis

Base calling was used to transform image data output from the sequencing machine into sequence data. These raw reads are stored in the fastq format. Raw reads contain dirty reads that contain adapters, unknown or low-quality bases. These data will negatively affect subsequent bioinformatic analyses. Thus, the dirty reads were filtered out to leave only clean reads. The data used in this publication have been deposited in the National Instituted of Health (NIH) Short Read Archive database (http://www.ncbi.nlm.nih.gov/sra) and are accessible through Sequence Read Archive (SRA) accession number SRP031770. The short reads assembling program, Trinity [43], was used to assemble the *de novo* transcriptome.

The sequences produced by Trinity are termed unigenes. Unigenes from multiple samples from the same species are then further processed by sequence splicing and redundancy removal by sequence clustering software to produce non-redundant unigenes that are as long as possible. Subsequently, the unigenes were clustered into gene families to produce two classes: clusters and singletons. Clusters have the prefix, CL, followed by the cluster ID. One cluster comprises several unigenes whose similarity to one another is more than 70%. Singletons were given the prefix unigene.

Blastx alignment (*E*-value < 1.0 × 10^−5^) between unigenes and protein databases, including Nr, Nt, Swiss-Prot, KEGG and COG, was performed. The sequence directions of unigenes were decided by the best aligning results from all these databases. If different databases generated conflicting results, to decide the sequence direction of the unigenes, a priority order of Nr, Nt, Swiss-Prot, KEGG and COG was adopted. When a unigene could not be aligned to any of the above databases, the software, ESTScan [23], was used to decide its sequence direction.

### 3.5. Unigene Function Annotation

Unigene sequences were initially aligned to protein databases, including NR, Swiss-Prot, KEGG and COG (*E*-value < 1.0 × 10^−5^) by Blastx, and then aligned to nucleotide databases Nt (*E*-value < 1.0 × 10^−5^) by Blastn. Proteins with the highest sequence similarity with the given unigenes were retrieved, along with their protein functional annotations. Through KEGG annotations, we obtained the pathway annotation for the unigenes. Unigenes were also aligned to the COG database to predict and classify the possible functions of the unigenes.

### 3.6. Unigene GO Classification

With the Nr annotation, the Blast2GO program [44] was used to obtain the GO annotation of the unigenes. After GO annotation was searched for every unigene, to understand the distribution of the functions of the genes in *P. australis* at the macro level, GO functional classification was performed for all unigenes using the WEGO software [45].

### 3.7. Protein CDS Prediction

Unigenes were first aligned by Blastx (*E*-value < 1.0 × 10^−5^) to protein databases in the priority order of Nr, Swiss-Prot, KEGG and COG. Unigenes that aligned to a higher priority database were not aligned to the remaining lower priority databases. Proteins whose ranks are highest in the blast results were then retrieved to analyze the CDSs of the unigenes. The CDSs were translated into amino sequences using the standard codon table. Thus, both the nucleotide sequences (5′–3′) and amino sequences of the unigene CDSs were acquired. ESTScan was used to scan unigenes that could not be aligned to any database, producing the nucleotide sequence (5′–3′) direction and the amino sequence of the predicted coding region.

### 3.8. Unigene Expression Difference Analysis

The calculation of differentially expressed genes used the FPKM method (fragments per kb per million fragments) [46]. A rigorous algorithm has been developed to identify genes that are differentially expressed between two samples [47]. To identify genes with differential expression between two samples, the threshold of the *p*-value in multiple tests was determined using the false discovery rate (FDR) method [48]. A threshold of *FDR* < 0.001 and an absolute value of log2 ratio > 1 were used to judge the significance of the differences in gene expression. DEGs were then subjected to GO functional analysis and KEGG pathway analysis. To select more specific DEGs, a threshold of *FDR* < 0.001 and an absolute value of log2 ratio > 2 were used to retrieve common DEGs in both comparisons of PA2T *vs.* PA2 and PA4T *vs.* PA4.

### 3.9. Gene Ontology Functional Enrichment Analysis for Differentially Expressed Genes

First, all the DEGs were mapped to terms in the GO database (http://www.geneontology.org/), and the numbers of genes annotated with each GO term were calculated to obtain a gene list and the numbers of genes for every assigned GO term. To identify significantly enriched GO terms among the DEGs, a hypergeometric test was used. The *p*-value calculating formula in this hypothesis test is:

(1) $$p=1-\sum_{i=0}^{m-1}\frac{\left(\begin{array}{c} M\\ i \end{array}\right)\left(\begin{array}{c} N-M\\ n-i \end{array}\right)}{\left(\begin{array}{c} N\\ n \end{array}\right)}$$

where *N* is the number of all genes with GO annotation; *n* is the number of DEGs in *N; M* is the number of all genes that are annotated to the certain GO terms; *m* is the number of DEGs in *M*. A calculated *p*-value after Bonferroni correction of ≤0.05 was used as the threshold. GO terms that fulfilled this condition were defined as significantly enriched in the DEGs in the context of the whole transcriptome background. The GO functional enrichment analysis was used to recognize the main biological functions of the DEGs, as well as to integrate the clustering analysis of the expression patterns to easily obtain the expression patterns of the DEGs annotated with GO terms.

### 3.10. KEGG Pathway Analysis for DEGs

Pathway enrichment analysis retrieves significantly enriched pathways associated with DEGs in the context of the whole transcriptome background. The formula for calculating the *p*-value is similar to that used in the GO analysis. The *Q*-value is defined as the FDR analog of the *p*-value. After multiple test corrections, we choose pathways with *Q*-values of ≤0.05 as significantly enriched in DEGs.

### 3.11. qRT-PCR Analysis of Potential Drought Response DEGs

RNAs from the leaves of PA2T, PA2T-1, PA2T-2, PA2T-3, PA2, PA4T, PA4T-1, PA4T-2, PA4T-3 and PA4 (three samples from each) were extracted with an RN03-RNApure High-purity Total RNA Rapid Extraction Kit (Spin-column, Aidlab, Beijing, China). The RNA was then precipitated with isopropanol. Purified and concentrated RNA was denatured, and the first strand cDNA of all the samples was synthesized using the aniScriptc DNA synthesis kit (Bio-Rad, Hercules, CA, USA). cDNA was then amplified in the CFX96TM Real-Time System (Bio-Rad, Hercules, CA, USA) with So Fast Eva Green Supermix (Bio-Rad, Hercules, CA, USA). The following PCR parameters were used: 50 °C for 2 min, 95 °C for 1 min; followed by 40 cycles of 95 °C for 10 s and 55 °C for 15 s. Three replicates were analyzed for each gene. The average threshold cycle (*C*_t_) was normalized to the expression of the 18S rRNA of *Paulownia* as an internal reference gene. The relative expression changes were calculated with the 2^−ΔΔ^*^C^*^t^ method. All the primers for qPCR analysis are shown in Table 4.

## 4. Conclusions

The results presented in the current study suggest that several genes from different metabolic pathways are regulated by drought stress in *P. australis*, thus highlighting the impact of water deficit on associated genes and signaling pathways. Some of these genes and pathways are not exclusively involved in the response to drought, but are associated with other abiotic stresses, such as cold and salt. These genes and pathways are potential targets for improving the tolerance of plants to stresses.

## Figures and Tables

**Table 1.** Overview of the sequencing and assembly of the transcriptome of *P. australis*.

Statistics of data production	PA2	PA4	PA2T	PA4T
Number of clean reads	65,271,332	66,045,998	67,261,140	66,439,124
Total nucleotides (nt)	5,874,419,880	5,944,139,820	6,053,502,600	5,979,521,160
Q20 percentage (%)	97.38%	97.42%	97.31%	97.36%
*N* percentage	0.00%	0.00%	0.00%	0.00%
GC percentage (%)	46.94%	46.21%	46.41%	46.16%
Contigs	PA2	PA4	PA2T	PA4T

Number of contigs	135,157	159,240	147,935	159,789
Total nucleotides (nt) in contigs	44,242,508	49,398,069	44,264,333	49,481,225
Average length of contigs (nt)	327	310	299	310
Length of N50 (bp)	555	472	447	477
Unigenes	PA2	PA4	PA2T	PA4T

Number of unigenes	78,254	93,351	86,152	97,980
Total nucleotides (nt) in unigenes	63,081,300	66,626,947	63,561,654	78,415,946
Length of N50 (bp)	1,425	1,243	1,285	1,413
Average length of unigenes (bp)	806	714	738	800

**All unigenes**				

Number of all unigenes	111,660			
Total nucleotides (nt) in all unigenes	113,092,718			
Length of N50 (bp)	1,667			
Average length of all unigenes (bp)	1,013			

**Table 2.** Statistical analysis of the biological process, cell component and molecular function of the differentially expressed genes (*DEG*s) in both the PA2T *vs.* PA2 and PA4T *vs.* PA4 comparisons.

Ontology	Class	Number of DGEs
Biological process	biological adhesion	2
	biological regulation	252
	carbon utilization	5
	cell proliferation	4
	cellular component organization or biogenesis	179
	cellular process	562
	death	35
	developmental process	169
	establishment of localization	213
	growth	31
	immune system process	69
	localization	218
	locomotion	1
	metabolic process	583
	multi-organism process	137
	multicellular organismal process	169

Biological process	negative regulation of biological process	50
	positive regulation of biological process	43
	regulation of biological process	223
	reproduction	91
	reproductive process	90
	response to stimulus	379
	signaling	76
	single-organism process	217

Cellular component	cell	637
	cell junction	31
	cell part	637
	extracellular matrix	1
	extracellular region	132
	macromolecular complex	72
	membrane	362
	membrane part	129
	membrane-enclosed lumen	3
	nucleoid	1
	organelle	498
	organelle part	236
	symplast	31

Molecular function	antioxidant activity	9
	binding	375
	catalytic activity	490
	electron carrier activity	33
	enzyme regulator activity	8
	molecular transducer activity	11
	nucleic acid binding transcription factor activity	16
	nutrient reservoir activity	8
	protein binding transcription factor activity	2
	receptor activity	3
	structural molecule activity	5
	transporter activity	107

**Table 3.** Statistical analysis of the Kyoto Encyclopedia of Genes and Genomes (KEGG) analysis of the *DEG*s in both the PA2T *vs.* PA2 and PA4T *vs.* PA4 comparisons.

No.	Pathway	*DEG*s genes with pathway annotation	Pathway ID
1	Metabolic pathways	46 (6.84%)	ko00710
2	Biosynthesis of secondary metabolites	282 (41.9%)	ko01100
3	Carbon fixation in photosynthetic organisms	26 (3.86%)	ko00910
4	Endocytosis	150 (22.29%)	ko01110
5	Glycerophospholipid metabolism	23 (3.42%)	ko00195
6	Ether lipid metabolism	28 (4.16%)	ko00630
7	Plant hormone signal transduction	15 (2.23%)	ko00904
8	Phenylpropanoid biosynthesis	18 (2.67%)	ko00250
9	Plant-pathogen interaction	20 (2.97%)	ko00030
10	Starch and sucrose metabolism	21 (3.12%)	ko00260
11	Glyoxylate and dicarboxylate metabolism	19 (2.82%)	ko00908
12	Nitrogen metabolism	13 (1.93%)	ko00944
13	Photosynthesis	31 (4.61%)	ko00940
14	Glycine, serine and threonine metabolism	16 (2.38%)	ko00945
15	Pentose phosphate pathway	10 (1.49%)	ko00905
16	Pentose and glucuronate interconversions	32 (4.75%)	ko00565
17	Zeatin biosynthesis	5 (0.74%)	ko00196
18	Alanine, aspartate and glutamate metabolism	20 (2.97%)	ko00040
19	Stilbenoid, diarylheptanoid and gingerol biosynthesis	5 (0.74%)	ko00943
20	Spliceosome	7 (1.04%)	ko00740
21	Cyanoamino acid metabolism	14 (2.08%)	ko00903
22	Diterpenoid biosynthesis	15 (2.23%)	ko00460
23	Limonene and pinene degradation	10 (1.49%)	ko00360
24	Flavone and flavonol biosynthesis	8 (1.19%)	ko00073
25	Flavonoid biosynthesis	7 (1.04%)	ko00920
26	RNA transport	11 (1.63%)	ko00906
27	Ascorbate and aldarate metabolism	12 (1.78%)	ko00941
28	Carotenoid biosynthesis	29 (4.31%)	ko00500
29	Glycolysis/Gluconeogenesis	2 (0.3%)	ko00942
30	Amino sugar and nucleotide sugar metabolism	34 (5.05%)	ko00564
31	ABC transporters	12 (1.78%)	ko00053
32	Brassinosteroid biosynthesis	35 (5.2%)	ko04144
33	Arginine and proline metabolism	4 (0.59%)	ko00591
34	Phenylalanine metabolism	5 (0.74%)	ko00909
35	Galactose metabolism	10 (1.49%)	ko00051
36	Fructose and mannose metabolism	2 (0.3%)	ko00902
37	Purine metabolism	10 (1.49%)	ko00052
38	Oxidative phosphorylation	10 (1.49%)	ko00330
39	Pyrimidine metabolism	3 (0.45%)	ko00960
40	Cutin, suberin and wax biosynthesis	2 (0.3%)	ko00603
41	Circadian rhythm: plant	3 (0.45%)	ko00950
42	Protein processing in endoplasmic reticulum	6 (0.89%)	ko00511
43	Ribosome biogenesis in eukaryotes	2 (0.3%)	ko00901
44	Riboflavin metabolism	6 (0.89%)	ko00350
45	Sulfur metabolism	2 (0.3%)	ko00966
46	Pyruvate metabolism	2 (0.3%)	ko00604
47	Lysine degradation	1 (0.15%)	ko00785
48	Terpenoid backbone biosynthesis	6 (0.89%)	ko00310
49	Cysteine and methionine metabolism	2 (0.3%)	ko00750
50	Other glycan degradation	5 (0.74%)	ko00600
51	RNA polymerase	3 (0.45%)	ko00130
52	Tyrosine metabolism	2 (0.3%)	ko00402
53	RNA degradation	10 (1.49%)	ko00520
54	Peroxisome	8 (1.19%)	ko04712
55	Isoflavonoid biosynthesis	2 (0.3%)	ko00670
56	Sesquiterpenoid and triterpenoid biosynthesis	10 (1.49%)	ko02010
57	Photosynthesis; antenna proteins	1 (0.15%)	ko03450
58	Sphingolipid metabolism	6 (0.89%)	ko00270
59	Linoleic acid metabolism	2 (0.3%)	ko00061
60	alpha-Linolenic acid metabolism	4 (0.59%)	ko00592
61	Inositol phosphate metabolism	4 (0.59%)	ko00480
62	mRNA surveillance pathway	6 (0.89%)	ko00900
63	Glutathione metabolism	3 (0.45%)	ko00400
64	Glycosylphosphatidylinositol (GPI)-anchor biosynthesis	3 (0.45%)	ko00563
65	Porphyrin and chlorophyll metabolism	2 (0.3%)	ko00531
66	Tropane, piperidine and pyridine alkaloid biosynthesis	3 (0.45%)	ko00860
67	Phenylalanine, tyrosine and tryptophan biosynthesis	1 (0.15%)	ko00760
68	Isoquinoline alkaloid biosynthesis	6 (0.89%)	ko03020
69	Citrate cycle (tricarboxylic acid (TCA) cycle)	5 (0.74%)	ko04146
70	Glycerolipid metabolism	31 (4.61%)	ko04075
71	Ubiquinone and other terpenoid-quinone biosynthesis	1 (0.15%)	ko00450
72	beta-Alanine metabolism	11 (1.63%)	ko00010
73	Ubiquitin mediated proteolysis	4 (0.59%)	ko00562
74	Natural killer cell mediated cytotoxicity	2 (0.3%)	ko03410
75	Fatty acid biosynthesis	3 (0.45%)	ko00410
76	Glycosphingolipid biosynthesis: ganglio series	2 (0.3%)	ko03030
77	Anthocyanin biosynthesis	9 (1.34%)	ko00190
78	Glycosaminoglycan degradation	1 (0.15%)	ko00770
79	Fatty acid metabolism	1 (0.15%)	ko00290
80	Monoterpenoid biosynthesis	9 (1.34%)	ko00240
81	Glucosinolate biosynthesis	2 (0.3%)	ko04650
82	Benzoxazinoid biosynthesis	7 (1.04%)	ko00620
83	DNA replication	1 (0.15%)	ko01040
84	Indole alkaloid biosynthesis	7 (1.04%)	ko03008
85	Tryptophan metabolism	3 (0.45%)	ko00020
86	Valine, leucine and isoleucine degradation	2 (0.3%)	ko00380
87	One carbon pool by folate	3 (0.45%)	ko00561
88	Vitamin B6 metabolism	31 (4.61%)	ko04626
89	Glycosphingolipid biosynthesis: globo series	1 (0.15%)	ko00650
90	Base excision repair	1 (0.15%)	ko03050
91	Selenocompound metabolism	6 (0.89%)	ko03018
92	Lipoic acid metabolism	9 (1.34%)	ko00230
93	Pantothenate and CoA biosynthesis	1 (0.15%)	ko00510
94	*N*-Glycan biosynthesis	2 (0.3%)	ko00280
95	Biosynthesis of unsaturated fatty acids	2 (0.3%)	ko00071
96	Butanoate metabolism	15 (2.23%)	ko03040
97	Basal transcription factors	1 (0.15%)	ko03022
98	Non-homologous end-joining	12 (1.78%)	ko03013
99	Phosphatidylinositol signaling system	4 (0.59%)	ko03015
100	Nicotinate and nicotinamide metabolism	1 (0.15%)	ko04070
101	Ribosome	7 (1.04%)	ko04141
102	Proteasome	3 (0.45%)	ko04120
103	Valine, leucine and isoleucine biosynthesis	1 (0.15%)	ko03010

**Table 4.** Primers of quantitative RT-PCR analysis of candidate drought response genes. “-f” represents forward primers, and “-r” represents reverse primers.

Potential gene function	Nr-ID	Size (bp)	Primer	Sequence
Ribulose bisphosphate carboxylase	gi|255582745|ref|XP_002532149.1|	952	CL10638.Contig1-f	AATACCTTCTCCGTCTCAAG
			CL10638.Contig1-r	TCGTCCAATTCGTTCACC
mitogen-activated protein kinase kinase	gi|290784293|gb|ADD62693.1|	217	CL11655.Contig1-f	GCGGAGGATGGAGACTTC
			CL11655.Contig1-r	AGTTCACCACAAGCACAC
laccase-14	gi|359495139|ref|XP_002264394.2|	988	CL14171.Contig2-f	CCAACCAACCACATAGAAG
			CL14171.Contig2-r	TTAACTACACGGCGGATG
Asparagine synthetase	gi|5915696|sp|O24661.3|ASNS_TRIVS	2,370	CL1834.Contig1-f	ATAAGGAGTTGAAGGAATGGC
			CL1834.Contig1-r	ACTTGATGGCTCTGTCTG
alanine--glyoxylate aminotransferase 2	gi|225434396|ref|XP_002270785.1|	2,078	CL1998.Contig8-f	ACAATAGCATCCACCACCTGAG
			CL1998.Contig8-r	CCGCCGTCTTCCACTTCTTC
dehydrin	gi|157497151|gb|ABV58322.1|	551	CL3830.Contig3-f	CCACAACACAAGACCACCAAC
			CL3830.Contig3-r	TCACTCCACCGCCACTCC
inositol oxygenase 1	gi|225442398|ref|XP_002282395.1|	899	CL5837.Contig1-f	CTATACAGCAAGAGCAAGGTTCGG
			CL5837.Contig1-r	TCCAAGCATCCAGCCATTTCAAG
carbonic anhydrase	gi|225452452|ref|XP_002277957.1|	1,384	CL5917.Contig2-f	TCCTCCTCCACTGACTTC
			CL5917.Contig2-r	CTTCACCAATCCATCTCTAAC
flavonoid glycosyltransferase	gi|260279128|dbj|BAI44134.1|	1,662	CL7275.Contig5-f	TTCTTCGTTCTCCATCTTCATC
			CL7275.Contig5-r	TGTTATCAGAGGCAGGTAGC
short chain alcohol dehydrogenase	gi|255565739|ref|XP_002523859.1|	1,722	CL9398.Contig3-f	AGGCGTAGGAACAAGGTATGG
			CL9398.Contig3-r	CATTGGTGGTGGTGCTTCG
cytochrome P450 76A2	gi|255539320|ref|XP_002510725.1|	1,762	Unigene1109-f	CTTCCTCAGTGTCAATCC
			Unigene1109-r	TCGCAGAATATGTGTTGG
geraniol 10-hydroxylase	gi|300193870|gb|ADJ68324.1|	1,796	Unigene11285-f	ATGTCCACTTCTGATTCC
			Unigene11285-r	GGTTCCATTCTTGATTCC
disease resistance response protein 206	gi|225441531|ref|XP_002280791.1|	864	Unigene11643-f	AACCAACCACCAGTAGAAGC
			Unigene11643-r	CCGAGTCCGAAGTGATTGC
photosystem I reaction center subunit X psaK	gi|325180223|emb|CCA14626.1|	597	Unigene4744-f	GCTCGCTGTGACTTCATTGG
			Unigene4744-r	TGCCTTCCTGTTCGCTGA
